# 

**Published:** 1848

**Authors:** 


### CONTENTS.

### PART I.—ORIGINAL COMMUNICATIONS.

ART. 1. Inflammation of the Vena Portæ (Pylephlebitis.) By Dr. John Waller, of Prague. Translated from the German, by Dr. Stahl, M.D., of Quincy, Illinois.
ART. 2. Intestinal Worms—their effects, &c., in the adult. By W. Matthews, M.D., of Putnam county, Indiana.
ART. 3. Retroversion and Anteversion of the Uterus. By John Evans, M.D., Professor of Obstetrics, &c., in Rush Medical College.

### PART II.—REVIEWS.

ART. 1. Elements of Pathology. By Alfred Stille, M.D., Lecturer on Pathology and the Practice of Medicine in the Philadelphia Medical Association, &c. Being No. 2 of the Medical Practitioner's and Student's Library.

### PART III.—BIBLIOGRAPHICAL NOTICES.

ART. 1. Females and their Diseases. By Charles D. Meigs, M.D., Professor of Midwifery, &c., in Jefferson Medical College.
ART. 2. Report of the Chicago Retreat for the Insane. By Edward Mead, M.D., Superintendent.

### PART IV.—SELECTIONS.

ART. 1. Code of Medical Ethics of the National Medical Association.
ART. 2. Letter from Professor J. A. Eve to the Editor of the Medical and Surgical Journal, on the use of Chloroform in Obstetric Practice.
ART. 3. On Euonymous Atropurpureus et Americanus. By Charles A. Santos, of Norfolk, Virginia.
ART. 4. Influence of Statistics on Quackery. Extract from Dr. Cartwright's Lecture.

### PART V.—EDITORIAL.

ART. 1. The Theory and Practice of Surgery. By the late Geo. McClellan, M.D.
ART. 2. Medical Ethics.
ART. 3. Medical Legislation.
ART. 4. Our Journal.
ART. 5. Western Medical Convention.
ART. 6. Medical Intelligence.

